# Anæsthesia and Anæsthetics

**Published:** 1859-01

**Authors:** 


### EDITORIAL DEPARTMENT.

*Anæsthesia and Anæsthetics.*—There is probably no subject, which is now actively agitated by the medical press, so important as that which heads this article. The use of anæsthetic agents is not confined to any speciality; it is used now by the general practitioner in tetanus, hydrophobia, convulsions and many other diseases; by the obstetrician, in labor and in obstetrical operations, and by the dentist. Those who practice surgery experience, its benefits every day. The discovery of agents which, for the time being, abolished all sensibility was justly hailed as one of the most useful advance, which science had ever made; and every American physician has been proud to say that his country gave it to the world. But a short time had elapsed after the patent taken out, by Drs. Jackson and Morton in 1846, for the discovery of the anæsthetic properties of either, when Prof. Simpson of Edinburgh announced the discovery of chloroform, which has, until very lately, entirely supplanted the use of ether in Europe. It was but a sho time ago, indeed, that chloroform was almost universally used in this country especially in the south and west.

We have lately noticed two able articles discussing the relative merits of anæsthetic agents; one in the *Boston Medical and Surgical Journal*, Dec. 9, 1858, and the other in the December No. of the *Medical News and Library*, extracted from the *London Lancet.* The first article is by Dr. Cotting, of Roxbury, and treats of the subject in its application to obstetrics. This view of the subject we shall not discuss at this time; but we feel interested to look at the opinion of distinguished members of our profession in regard to the comparative safety of ether and chloroform. Every one knows that in Philadelphia, Boston, New York and their vicinities, physicians fear chloroform, and substitute ether in nearly all cases. We see by the reports of the clinics at the Jefferson School, that Prof. Gross has not been convinced of the danger of the former agent, and continues to administer it before the class, in spite of the strong feeling which exists against it in Philadelphia. It is too true that our warnings in regard to the use of chloroform have, of late years, been fearfully frequent, and there is every reason to suppose that many deaths have occured under its administration which have never found their way into the journals. By the late article in the *London Lancet* by Dr. Glover, of Edinburgh, we learn that the first fatal case, resulting from the administration of chloroform, was reported in the *Lancet* for Feb. 5, 1848; the case was as follows:

"The girl had been operated upon for onychia, in the Newcastle Infirmary, on the 24th of October. The left great toe-nail was then successfully removed *under the influence of ether.* On the 28th, Mr. Meggison proceeded to remove the nail of the great toe of the right foot for the same disease. This, the first fatal case, is so instructive, that it deserves to be specially recorded. Mr. Meggison's statement was as follows: "I seated her on a chair, and put about a teaspoonful of chloroform into a tablecloth and held it to her nose. After she had drawn her breath twice, she pulled my hand down. I told her to draw her breath naturally, which she did, and in about half a minute I observed the muscles of the arm become rigid and her breathing a little quickened, but not stertorous. I had my hand on her pulse, which was natural until the muscles became rigid; it then appeared somewhat weaker—not altered in frequency. I then told my assistant, Mr. Lloyd, to begin the operation, which he did, and took the nail off. When the semicircular incision was made, she gave a struggle or jerk, which I thought was from the chloroform not having taken sufficient effect. I did not apply any more. Her eyes were closed, and I opened them, and they remained open; her mouth was open; and her lips and face blanched. When I opened her eyes they were congested. I called for water when I saw her face blanched, and I dashed some of it in her face. It had no effect. I then gave her some brandy, a little of which she swallowed with difficulty. I then laid her down on the floor, and attempted to bleed her in the arm and jugular vein, bnt only obtained about a spoonful. She was dead, I believe, at the time I attempted to bleed her. The last time I felt her pulse was immediately previous to the blanching appearance coming on, and when she gave the jerk. The time would not be more than three minutes from her inhaling the chloroform.' These are the exceedingly well described external symptoms of many cases of chloroform poisoning. Mr. Lloyd confirmed Mr. Meggison's statement.

"The inquest was adjourned from the 29th of January to the 1st of February to admit of a post-mortem examination, and a report on the case by Sir John Fife and myself, who were employed for the purpose.

"We presented a report, detailing the post-mortem examination, and giving our conclusions. We found the chief morbid appearances in the respiratory organs. 'There was great congestion of the lungs, which did not collapse, and were mottled with patches of a deep purple, bluish, or scarlet hue; the pulmonary tissue was filled with bloody froth, which was also found in the interior of the bronchi, mixed with mucus. On examining the larynx and trachea, the epiglottis was observed to be reddened at the summit, and of a vermilion hue.' The heart contained dark fluid blood in both its caviities, very little in the left; the membranes of the brain were somewhat congested.

"The jury returned the following verdict in accordance with our evidence: 'We are unanimously of opinion that the deceased, Hannah Greener, died from congestion of the lungs, from the effects of chloroform, and that no blame can be attached to Mr. Meggison, surgeon, or his assistant, Mr. Lloyd.' I had stated that I had analyzed the chloroform, and found it to be pure; and that, in my opinion, *there would generally be found more danger from the use of chloroform in slight operations, than in serious ones.* This bold and unhesitating opinion of Sir John Fife and myself, at a time when all was *couleur de rose* with regard to chloroform, naturally caused a great sensation both amongst the public and in the medical world."

It would have seemed that this solemn warning would have put a stop to the free and fearless use of chloroform; but the inventor of, and the advocates for an agent, for which so much was claimed, would not be intimidated by this accident; and Dr. Simpson asserted boldly, that the death was not due to the administration of chloroform, but to strangling, produced by the brandy and water which was administered to recover the patient. Repeated instances of death, however, placed the occasional fatal power of chloroform beyond a doubt; and now, no one who pays the slightest attention to the literature of this subject, can doubt that death has frequently occurred during its administration. Not only has death occurred, but it has happened to those must skilled and experienced in its use. We were educated in the chloroform school. Prof. Gross, now of the Jefferson School, at Philadelphia, and Prof. Miller, lately the able Professor of Obsterics in the University of Louisville, under whom we had the honor to sit, were in the constant habit of administering, and of recommending chloroform. Professor Hamilton, however, has always given it with some reluctance, but which was much less in former years than it is now. Our prejudices, then, were decidedly in favor of its use; but now, our views are changed, and we believe that Prof. Hamilton is becoming more and more convinced of the dangers of producing anæsthesia by chloroform, and so teaches the class that sit yearly under his instruction. We have ourselves seen patients who appeared to be carried by chloroform to the very threshhold of the grave, and though we have administered it in our own practice, and at the request of other surgeons, we have latterly experienced a horrible foreboding of evil while the patient was under its influence, and an inexpressible relief, when its effects passed off. Still we have administered it, and very lately, but have nearly come to the conclusion that we will never do so again.

A friend has sent us an extract from an address read before the *British Medical Association* by Prof. Miller, of Edinburgh, which indicates the apprehensions which are now entertained in regard to the use of chloroform, by many of the most eminent European practitioners. We have not the space to copy the entire extract, but will merely quote a passage referring directly to the subject before us. Prof. Miller, in the first place, mentions that the danger of its administration is less in young children than in adults. He adds:

"It becomes me to take this opportunity of stating, that when chloroform was first brought into use here, I was a great enthusiast in its praise. I esteemed it to be as safe and manageable as it was beneficial, and thought that it could hardly be used too often or too freely. I committed these thoughts to written words; and as I wrote, my pen warmed with an honest enthusiasm. But subsequent events have chastened and subdued my tone; and my present 'surgical experience of chloroform' amounts to this: never to withhold its use when that use seems to be imperatively required; and never to sanction its use unless this seems to be absolutely demanded; always conducting its administration with the utmost prudence and caution, so as to avoid those terrible results which errors or excess, even under the protection of an existing tolerance, may not fail to produce, and from which, as experience has too fully shown, even faultless administration is not altogether free. Into the question as to whether ether or chloroform be the safer anæsthetic, I do not enter. Both are imperfect; and ere long an agent superior to both may be discovered. Meanwhile, let us be thankful for what we have, using it with wise economy and care; and let us mark off an advance in this direction as one of the true radii formerly alluded too as suitable and safe for the surgical pioneer."

The mode of death from inhaling this agent is very peculiar; its fearful character is due to its suddenness, resulting while we suppose the patient to be entirely safe, and the utter futility of all our efforts to give relief. The case which we have quoted, which was the first case of death reported, is an excellent example. The pulse suddenly is lost, two or three labored inspirations, and all is still; our patient is dead. We have never witnessed such a fearful scene, and we earnestly pray that it may never occur to us, or to those whose operations we may witness. The post-mortem morbid appearances are generally confined to the lungs, which are found highly congested, and almost solidified; this is very peculiar and intense, and from the descriptions, we should say that it resembled the post-mortem appearances of the lungs, after both pneumo-gastric nerves had been divided. After the division of both pneumo-gastric nerves, in the dog, for example, we have death resulting in a few hours, accompanied by a gradual diminution in the frequency of the respirations; it is unnecessary here to dwell upon the peculiarities of this respiration, suffice it to say that the appearances of the lungs present a peculiarity somewhat similar to those described in cases of death from chloroform; they are almost carnified, have lost their crepitation, and are quite black. The difference between these appearances and those attendant upon death from chloroform, might be due to the immediate death in the one instance, and the hours which elapse before a fatal result in the other. In a dog, on which we performed the experiment of dividing both pneumo-gastrics before the class of the Buffalo Medical College, but a few weeks since, we had the morbid appearances which we have described; life had been prolonged for about twenty hours.

The cause of death, in these fatal cases, is uncertain. It is sometimes said that the influence of the anæsthetic agent, is exerted first on the cerebrum, and from thence, when it is pushed too far, it extends to the medulla oblongata; respiration is of course arrested, and death ensues. We imagine, however, that the cause of death will always be conjectural, but whatever it may be, it is certain that death frequently occurs under the use of chloroform.

We will now look into the comparative merits and advantages of the sulphuric ether. Ether is less prompt in its action than chloroform; it is more disagreeable both to the patient and operator; sometimes it is quite difficult to produce anæthesia; and its after effects, as nausea, giddi ness, etc., are more severe. These disadvantages have led to the general use of chloroform, and it is undoubted that the latter is much preferable in these respects. But the foregoing facts make it necessary for us to compare them in another point of view. Which is the more hazardous to the life of the patient? In 1848, Thomas H. Wakely, Surgeon to the Royal Free Hospital, performed an hundred experiments with ether and chloroform on dogs; these experiments have been referred to in the article by Dr. Glover, which we have quoted. The symptoms produced by these agents are very similar, but chloroform is "*assuredly the more dangerous one of the two.*" Those who have been accustomed to use these two agents in vivisections, have all remarked that death is very likely to occur in administering chloroform, even when it is done with the greatest skill and care; a like result seldom follows the administration of ether, with which agent it is frequently almost impossible to kill a dog; though we have once seen such an instance. We think that there is no case of death in the human subject resulting from the use of ether, with the exception of a single one, in which it was easily attributable to other causes. Cases of death in the lower animals, from chloroform, are extremely freqnent, while in man, they are, no doubt, quite rare; and it is, therefore, the general opinion, and apparently a just one, that the lower animals are much more the easily killed by an anæsthetic agent. If, therefore, it be *almost* impossible to kill a dog with ether, it would be *quite* impossible to kill a human being, provided there were no direct contra-indication to anæsthesia.

We have no intention of going into a comparison of other means of producing anæsthesia; the great issue is now between chloroform and ether. All are familiar with the history of amylene, which was soon abandoned by the profession, in consequence of fatal cases occurring even in the hands of its distinguished discoverer, Dr. Snow. There have been various means proposed for the production of local anæsthesia, such as electricity, compression, carbonic acid, or a freezing mixture. These means have never been generally employed. We have another substance, *acétone*, which has been lately proposed by Mr. Béchamp of Paris; this is said to be rapid and effectual in its action, and, with it, that it is impossible to kill so delicate an animal as a rabbit. If this assertion be confirmed, *acétone* will undoubtedly take the first rank among anæsthetic agents; but the operations of amylene were described at first in the same way.

But to return to ether and chloroform, what are the general conclusions to which we must arrive, after a careful review of the whole subject? We conceive that we cannot but regard chloroform as an agent, often beautiful and satisfactory in its action, bnt possessing a fearful power, which may be developed in any case, and under any circumstances. Accidents are irrespective of any peculiarity of constitution which we can discover; they are liable to occur in the most experienced and skillful hands; and sometimes after the administration of but a very small quantity of the article. Ether, on the other hand, less prompt and agreeable in its action, is almost as effectual as chloroform, and *absolutely without danger to life.* These statements being *facts,* we fear that we must repudiate chloroform, and use ether. We have no right, in a matter which affects the life of a human being, to shut our eyes to truths, be they as unwelcome as they may.

